# Assessment of *Escherichia coli*-derived Recombinant Human Bone Morphogenic Protein-2 on Fertilization and Early Embryonic Development in Rats

**DOI:** 10.1007/s11095-023-03514-z

**Published:** 2023-04-20

**Authors:** Nam Hyun Kim, Seul Ki Min, Myeong Wook Lee, Seung-Hoon Kang

**Affiliations:** 1 Life Science Institute, Daewoong Pharmaceuticals, 72, Dugye-Ro, Pogok-Eup, Cheoin-GuGyeonggi-Do, Yongin-Si, 17028 Republic of Korea; 2 Department of Agricultural Biotechnology, Seoul National University, Seoul, Republic of Korea

**Keywords:** early embryonic development, fertility, rat, reproductive toxicity, rhBMP-2

## Abstract

**Objective:**

Bone morphogenetic protein-2 (BMP-2) impacts fertility in women by affecting the menstrual cycle and embryonic development. We aimed to determine the reproductive toxicity of *Escherichia coli* (*E. coli*)*-*derived recombinant human BMP-2 (rhBMP-2) by measuring changes in the reproductive performance and organs in rhBMP-2-treated rats.

**Methods:**

Overall, 88 male and female rats each were categorized into one control and three experimental groups. rhBMP-2 was intravenously administered to the experimental groups at 0.05, 0.15, and 0.50 mg/kg/day, respectively. The male rats were administered rhBMP-2 daily, starting from 28 days before mating until the day of necropsy (48 days), after which they were euthanized and necropsied. The female rats were administered rhBMP-2 daily, starting from 14 days before mating until 7 days after fertilization (22–36 days), after which they were necropsied 13 days after fertilization.

**Results:**

No rhBMP-2-related death occurred throughout the study period. All rhBMP-2-treated groups showed swelling in the tail at the site of rhBMP-2 administration. In the high-dose rhBMP-2 group, the male rats showed a slight reduction in body weight and food consumption, whereas the female rats showed a reduction in the weights of the ovary and oviduct. Examining the fertilization status and necropsy showed no effect of rhBMP-2 on fertility and early embryonic development. The no-observed-adverse-effect level of rhBMP-2 was 0.50 mg/kg/day in all rats.

**Conclusion:**

rhBMP-2 had no reproductive toxicity on the reproductive performance and organs in female and male rats. Therefore, these results provide new toxicology information on *E. coli*-derived rhBMP-2 as a therapeutic protein.

## Introduction

Bone morphogenetic protein-2 (BMP-2) can promote bone growth and differentiation among all biological proteins [1, 2]. BMP-2 is a growth factor that belongs to the transforming growth factor beta superfamily that impacts the reproductive function of various organisms (3). Recombinant human BMP-2 (rhBMP-2) is commonly used in spinal fusion, degenerative disc disease, and dentistry (4, 5). rhBMP-2 has been widely used in spine fusion procedures since its approval by the Food and Drug Administration (FDA), and various side effects of rhBMP-2 have been reported (6, 7). The most recognized clinical side effect of rhBMP-2 is an ectopic bone formation. Moreover, bone cyst formation and inflammatory compositions are commonly reported side effects of rhBMP-2 (8–11). Although rhBMP-2 can affect reproductive function, clinical studies related to the reproductive toxicity of rhBMP-2 have not been conducted. Therefore, using commercial rhBMP-2 products is prohibited in pregnant women (5).

Chinese hamster ovary (CHO) cell-derived recombinant proteins have been widely used to treat cancer, human immunodeficiency virus, and other diseases. However, they have limitations, such as high manufacturing costs and low production yield. *Escherichia coli (E. coli)*-derived proteins have been used as an alternative for many research and clinical applications due to the high production yield (12–16). In the case of *E.coli*-derived rhBMP-2, a structural difference exists based on the presence or absence of post-translational glycosylation. Despite these structural differences, several studies have reported that *E.coli*-derived rhBMP-2 and CHO cell-derived rhBMP-2 showed similar biological activities (14–16).

BMP-2 regulates the menstrual cycle and embryonic development and can affect fertility in women (17, 18). Moreover, BMP-2 regulates the activity of the follicle-stimulating hormone receptor and luteinizing hormone receptor via the Suppressor of Mothers Against Decapentaplegic (SMAD) pathway, thereby affecting folliculogenesis and oogenesis in women (19). In addition, BMP-2 is involved in gastrulation and amnion/chorion development via the SMAD pathway and consequently affects embryonic development (20–22).

Therefore, this study investigated the reproductive toxicity of *E. coli*-derived rhBMP-2 on the fertility and early embryonic development of rats and determined the safety margin of rhBMP-2 before its commercialization (segment I). Furthermore, *in vivo* research on the reproductive toxicity of rhBMP-2 can provide preclinical data to verify the safety of the clinical dose of rhBMP-2.

## Materials and Methods

The study was conducted at the Charles River Laboratories (Ashland, OH, USA) in accordance with FDA standards and the Organisation for Economic Co-operation and Development guidelines for testing at a nonclinical laboratory satisfying the Good Laboratory Practice standards. This study was reviewed and approved by the Institutional Animal Care and Use Committee before it was conducted (approval date: October 14, 2020). In addition, this study was conducted according to the Final Rules of the Animal Welfare Act regulations (Code of Federal Regulations, Title 9) and Public Health Service Policy on Humane Care and Use of Laboratory Animals (Office of Laboratory Animal Welfare, 2015). The study design adhered to the International Council for Harmonisation (ICH) guideline S5 (R3) (23).

### Test Substance

The test article, rhBMP-2 and control, formulation buffer, were obtained from the Bio R&D center Daewoong Pharm. Co., Ltd. The purity of rhBMP-2 was 96%, which was determined using reverse-phase high-performance liquid chromatography. Impurities corresponding to 4% are the oxidized and deamidation form of rhBMP-2. A formulation buffer of rhBMP-2 was used as a control containing L-glutamic acid (3.7 g/L), glycine (25 g/L), sucrose (5 g/L), polysorbate 80 (0.1 g/L), and sodium chloride (0.1 g/L) in water.

### Animals and Husbandry

Male and female Sprague–Dawley (SD) rats (Crl:CD, Charles River Laboratories, Inc., Raleigh, NC) were used in this study. The males and females were approximately 9 and 11 weeks old, respectively, and weighed between 193 and 249 g for males and 190 g and 242 g for females at the initiation of dosing. Crl:CD (SD) rats were acclimated before dosing. The males and females were assigned to four groups by a stratified randomization scheme designed to achieve similar group mean body weights (n = 22 per group/sex). Environmental conditions were set to maintain a relative humidity of 30–70% and a temperature of 20–26 °C, and the light cycle was maintained to provide a 12-h light/dark cycle.

### Experimental Design and Article Administration

**Fig. 1:**
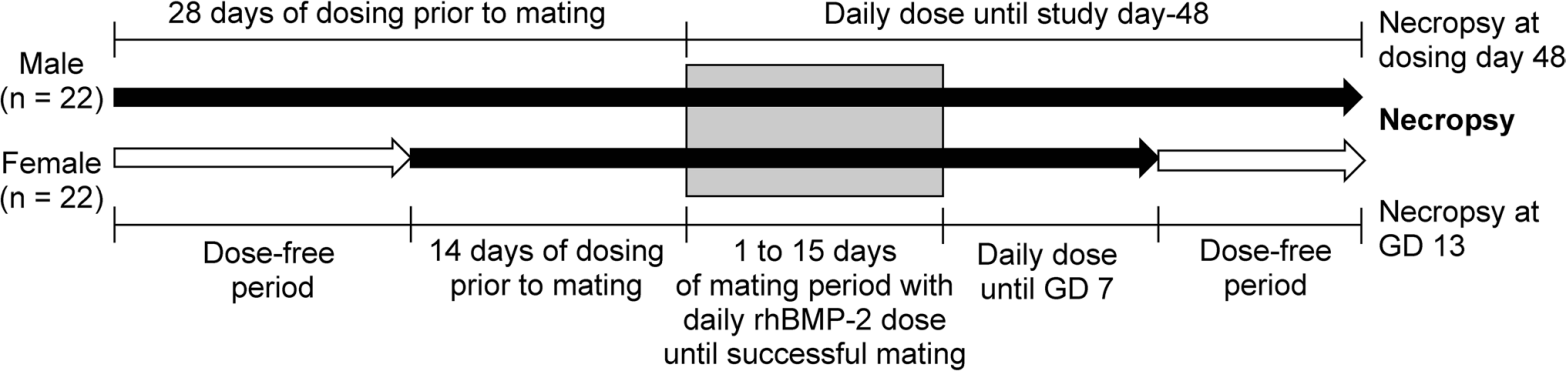
Experimental process design GD, gestation day. rhBMP-2, recombinant human bone morphogenetic protein-2. All four experimental groups were performed under the same experimental design. Black arrow: dosing period, white arrow: dose-free period

The experimental process is shown in Fig. 1. Among the four groups, 0.05, 0.15, or 0.5 mg/kg/day rhBMP-2 was administered to three groups, respectively, and the vehicle was administered to one group, which served as the control group. The test article and vehicle were administered as a single daily intravenous (bolus) injection to the tail vein. rhBMP-2 doses of 0.05, 0.15, and 0.5 mg/kg/day were administered daily in males starting from 28 days before mating until euthanasia. For females, rhBMP-2 doses of 0.05, 0.15, and 0.5 mg/kg/day were administered daily, starting from 14 days before mating until gestation day (GD) 7. Females without evidence of mating were dosed until the day prior to euthanasia, which is study day 48. These females were confirmed to be pregnant at necropsy, although they did not show evidence of mating (vaginal plug) during the mating period.

### Observation and Measurements

Clinical signs of toxicity, moribundity, and mortality were monitored daily. Observed clinical signs were recorded and reported in detail. The body weights of the males were recorded two times weekly after rhBMP-2 administration (i.e., every 3 and 4 days) until euthanasia. Similarly, the body weights of the females were recorded two times weekly after rhBMP-2 administration (i.e., every 3 and 4 days) until copulation and on GD 0, 3, 7, 10, and 13, or until euthanasia for females with no evidence of mating.

Estrous cycling was measured in females during the 2-week premating and mating periods until evidence of mating was observed. The males were euthanized on day 48, whereas the females were euthanized on GD 13 through carbon dioxide inhalation and subsequently necropsied. The following organ weights were measured: the brain, epididymides, ovary, and testes. Moreover, a microscopic examination was conducted on the epididymides, gland, ovary, oviduct, site of administration, testis, uterus, and vagina. Representative samples of tissues were collected and preserved in 10% neutral buffered formalin.

### Examination of Reproductive Function

Females were paired 1:1 with a male for mating for a maximum of 15 days. A positive sign of successful mating was a copulatory plug or sperm detected in the vagina. After confirmation of mating, the female was returned to its cage, and the day was designated as GD 0. If no evidence of mating was observed after 10 days, the female was matched with another male for an additional 5 days. The mating, fertility, and pregnancy indices were calculated based on these results. The testes and epididymides were removed during the necropsy and weighed.

### Statistical Analysis

All data were expressed as mean ± standard deviation, except for reproductive function data. Body weight changes and organ weights were first analyzed using Levene’s test. If the variance of quantitative data was homogeneous, the one-way analysis of variance F-test was used; otherwise, the Kruskal–Wallis test was used. Subsequently, pairwise comparisons were conducted using Dunnett’s and Dunn’s tests after the F-test and Kruskal–Wallis test, respectively. All statistical tests were considered at a 5% significance level using JMP®16 (SAS Institute Inc.).

## Results

### Clinical Signs and Mortality

Two male rats were euthanized during the study period, each from the 0.15 and 0.50 mg/kg/day groups. The male rat in the 0.15 mg/kg/day group was euthanized on day 45. Its tail showed severe swelling, was bent, and turned black on days 29, 41, and 43, respectively. Since further administration in the tail was considered difficult, the rat was euthanized. In contrast, the rat in the 0.50 mg/kg/day group was euthanized on day 31. The rat showed swelling and black discoloration in the tail at the administration site on day 28. A scab was also observed on its tail on day 30. In addition, its body weight was reduced by 17 g between days 24 and 31.

An rhBMP-2 dose-dependent increase in tail swelling was observed throughout the administration period (data not shown). In male rats, tail swelling was observed in all rhBMP-2-treated groups. However, tail swelling was observed from day 42 in the 0.05 mg/kg/day group, which was the lowest concentration group, but it was observed from day 29 in most rats in the two groups with higher rhBMP-2 concentrations (i.e., 0.15 and 0.50 mg/kg/day). In female rats, tail swelling was observed from day 28, and the effect was dose-dependent. Tail swelling was observed in seven rats in the 0.05 mg/kg/day group, whereas those in the 0.15 and 0.50 mg/kg/day groups displayed swollen tails. In addition, five rats in the 0.50 mg/kg/day group had bent tails.

### Body Weight Changes

**Fig. 2:**
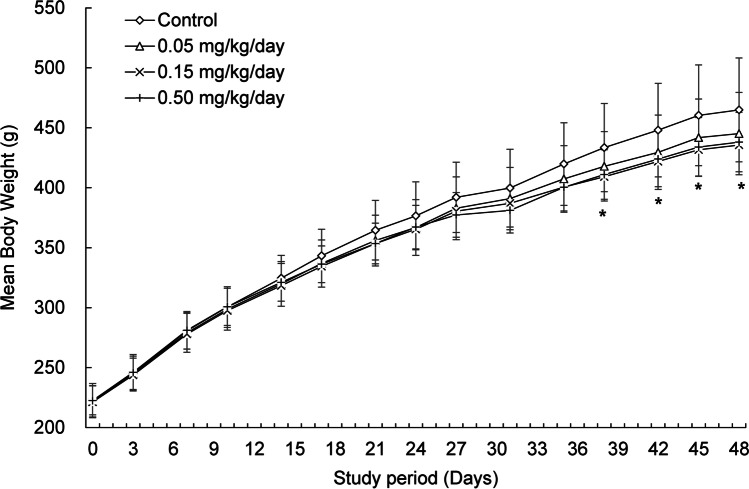
Mean body weights of the male rats treated with rhBMP-2 at 0, 0.05, 0.15, and 0.50 mg/kg/day. The weights of the 0.15 and 0.50 mg/kg/day groups significantly decreased with respect to that of the control from days 38 to 48 (days 38, 42, 45, and 48). The values are presented as mean ± standard deviation. * indicates a significant difference of p < 0.05 compared with the control group

**Fig. 3:**
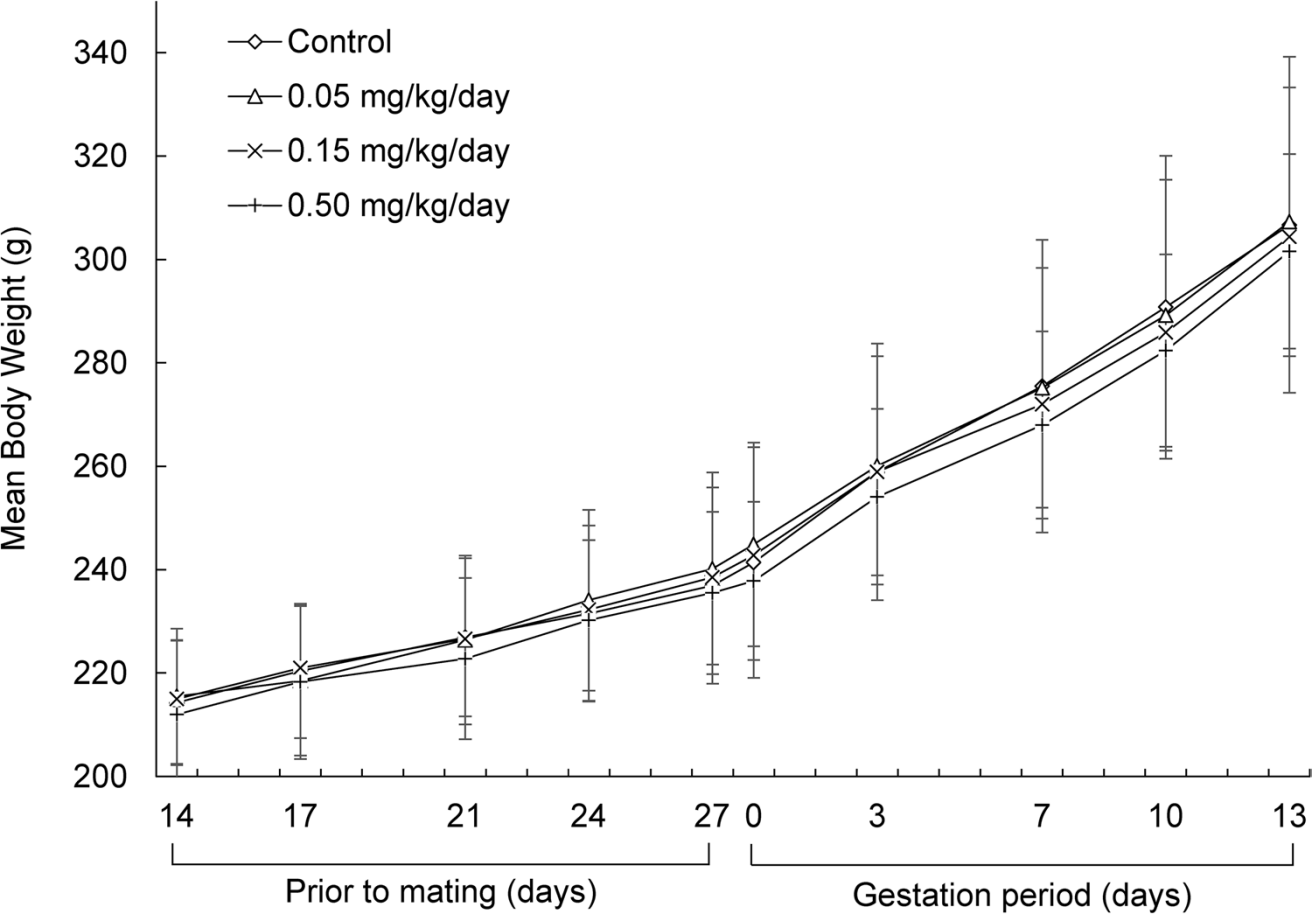
Mean body weights of the female rats treated with rhBMP-2 at 0, 0.05, 0.15, and 0.50 mg/kg/day**.** The values are presented as mean ± standard deviation

The male rats in the 0.05 mg/kg/day group and female rats in all rhBMP-2-treated groups did not show any rhBMP-2-induced weight changes (Figs. 2 and 3). As shown in Fig. 2, the male rats in the 0.15 and 0.50 mg/kg/day groups showed slight sporadic reductions in the mean weight gain compared with that of the control group. The weight of each rhBMP-2-treated group significantly decreased from 3.6% to 6.3% compared with that of the control group from days 38 to 48 (Fig. 2). Furthermore, the terminal body weights of the male rats in the 0.15 and 0.50 mg/kg/day groups were significantly decreased compared with that of male rats in the control group (Table I). The 0.15 and 0.50 mg/kg/day groups experienced 5.6–6.3% and 5.2–5.8% weight loss, respectively, compared with that of the control. However, slight differences in weight loss were observed with rhBMP-2 administration for the control, but it did not occur dose-dependently.

**Table I:** Organ Weights in the Mm ale Rats Treated with rhBMP-2

Parameter	rhBMP-2 dosage (mg/kg/day)^**a**^			
	0.00	0.05	0.15	0.50
Terminal Body Weight (g)	464.9 ± 43.3	445.2 ± 34.3	435.7 ± 24.8^*^	438.1 ± 37.1^*^
Brain (g)	2.07 ± 0.07	2.05 ± 0.09	2.06 ± 0.10	2.08 ± 0.11
Epididymis, Left (g)	0.64 ± 0.05	0.61 ± 0.05	0.60 ± 0.06	0.60 ± 0.05
Per Brain Weight (%)	30.70 ± 2.13	29.51 ± 2.45	29.17 ± 3.06	28.85 ± 1.83
Epididymis, Right (g)	0.65 ± 0.05	0.61 ± 0.06	0.61 ± 0.06	0.61 ± 0.05
Per Brain Weight (%)	31.22 ± 2.05	29.81 ± 2.80	29.42 ± 2.94	29.34 ± 2.57
Testis, Left (g)	1.82 ± 0.13	1.76 ± 0.12	1.78 ± 0.12	1.75 ± 0.12
Per Brain Weight (%)	87.78 ± 6.40	85.83 ± 6.18	86.36 ± 6.45	84.36 ± 5.93
Testis, Right (g)	0.65 ± 0.05	0.61 ± 0.06	0.61 ± 0.06	0.61 ± 0.05
Per Brain Weight (%)	88.55 ± 6.04	85.68 ± 6.66	85.75 ± 7.24	84.34 ± 5.44

^a^Values are presented as mean ± standard deviation

^*^ indicates a significant difference of p < 0.05 compared with the control group

rhBMP-2, recombinant human bone morphogenetic protein-2

### Gross Findings

The two male rats, each from the 0.15 and 0.50 mg/kg/day groups that were euthanized on days 45 and 29, respectively, showed a scab at the injection site. The rat in the 0.50 mg/kg/day group showed a multifocal focus and black discoloration in addition to the scab. However, no unusual necropsy and gross findings related to rhBMP-2 administration were observed in the surviving male and female rats. These changes were not test article-related because they were limited to the injection site and were the common findings in laboratory rats receiving tail vein injections.

### Organ Weights

For male rats, no significant differences were found between the rhBMP-2-treated and control groups for the weights of the brain, epididymis, and testis. In addition, no significant differences were observed in the organ-to-brain weight ratio for both sides of the epididymis and testis between the rhBMP-2-treated and control groups (Table I). The female rats in the 0.50 mg/kg/day group showed a significant reduction in the weight of the ovary and oviduct compared with that of the control (0.12 ± 0.02 vs 0.15 ± 0.02) (Table II). However, no effect on fertility associated with the observed weight reductions was observed, indicating no toxic effect of rhBMP-2 on fertility (Table III).

**Table II:** Organ Weights in the Female Rats Treated with rhBMP-2

Parameter	rhBMP-2 dosage (mg/kg/day)^a^			
	0.00	0.05	0.15	0.50
Terminal Body Weight (g)	306.7 ± 15.8	307.3 ± 17.9	304.4 ± 12.3	301.6 ± 20.2
Brain (g)	1.96 ± 0.09	1.97 ± 0.09	1.98 ± 0.07	1.97 ± 0.10
Ovary/Oviduct (g)	0.15 ± 0.02	0.14 ± 0.02	0.14 ± 0.02	0.12 ± 0.02^**^
Per Brain Weight (%)	7.64 ± 1.03	7.09 ± 0.81	7.10 ± 0.98	6.19 ± 0.88^**^

^a^Values are presented as mean ± standard deviation

^**^ indicates significant difference of p < 0.01 compared with the control group

rhBMP-2, recombinant human bone morphogenetic protein-2

**Table III:** Summary of the Reproductive Performance of Both Sexes of Rats Treated With rhBMP-2

Parameter	rhBMP-2 dosage (mg/kg/day)			
	0.00	0.05	0.15	0.50
Males				
Number of males	22	22	22	22
Mated males	22	19	21	22
Male impregnated a female	22	19	21	21
Pregnancy index (%)	100.0	86.4	95.5	95.5
Female				
Number of females	22	22	22	22
Mated females	22	22	22	22
Pregnant female	22	22	22	21
Pregnancy index (%)	100.0	100.0	100.0	95.5
Mean estrous cycle length	4.1	4.4	4.19	4.17

rhBMP-2, recombinant human bone morphogenetic protein-2

### Reproductive Performance

As shown in Table III, zero, three, one, and one male rats in control, 0.05, 0.15, and 0.50 mg/kg/day groups, respectively, failed to mate and become pregnant, whereas all female rats, except for one in the 0.50 mg/kg/day group, were successfully impregnated. Although the female rat in the 0.50 mg/kg/day group was not impregnated, mating was successful. No changes in mating and impregnation were observed in the rhBMP-2-treated groups, except for these cases. Based on the reproductive parameters (i.e., pregnancy index and estrous cycle length), rhBMP-2-induced changes in reproductive performance were not observed in both male or female rats. The female rats also showed no significant changes in their menstrual cycles (Table III).

### Cesarean Section Data

None of the rhBMP-2-treated groups showed changes in the indices of reproductive performance, including the number of pregnant females and the mean number of corpora lutea, implantations, and live embryos, compared with those of the control (Table IV). The 0.05, 0.15, and 0.50 mg/kg/day groups showed a decrease of 1.7%, 6.4%, and 12.7%, respectively, in the number of corpora lutea compared with those of the control. Although an rhBMP-2 dose-dependent change in the number of corpora lutea was observed, it was not significant (17.3 ± 2.5 vs 17.0 ± 3.1 vs 16.2 ± 2.3 vs 15.1 ± 2.5). Furthermore, no significant changes were observed for other indices, indicating that rhBMP-2 did not affect reproductive performance (Table IV).

**Table IV:** Summary of Ovarian and Uterine Examinations of the Female Rats Treated with rhBMP-2 During the Pre-mating and Early Gestation Periods

Parameter	rhBMP-2 dosage (mg/kg/day)^a^			
	0.00	0.05	0.15	0.50
Number of corpora lutea	17.3 ± 2.5	17.0 ± 3.1	16.2 ± 2.3	15.1 ± 2.5
Number of implantation	15.4 ± 2.1	13.9 ± 3.5	14.2 ± 3.3	13.7 ± 3.1
Pre-implantation loss (%)	10.4 ± 7.4	18.5 ± 18.3	13.5 ± 15.6	9.7 ± 13.9
Number of resorbed fetuses	0.5 ± 0.7	0.6 ± 0.9	0.7 ± 1.2	0.6 ± 0.8
Number of live embryo	14.9 ± 2.3	13.3 ± 3.6	13.5 ± 3.4	13.1 ± 3.1
Number of dead fetuses	0.0 ± 0.0	0.0 ± 0.0	0.0 ± 0.0	0.0 ± 0.0
Post-implantation loss	3.8 ± 5.4	5.1 ± 9.4	5.6 ± 10.3	4.9 ± 6.3

^a^Values are presented as mean ± standard deviation

rhBMP-2, recombinant human bone morphogenetic protein-2

## Discussion

Although the CHO-derived recombinant proteins have been mainly used in clinical applications for cancers and other diseases, alternative methods are needed because of the high production cost and low yield of the existing methods. Therefore, *E. coli* was suggested as an alternative source for the high-yield production of therapeutic proteins. This study examined the harmful effect of the reproductive toxicity of *E. coli*-derived rhBMP-2 on the fertility and early embryonic development of rats after intravenous administration during pre-mating and early gestation periods.

In current clinical practice, rhBMP-2 is implanted with an implant material to reduce initial burst release and maintain sustained release from the implantation site (23). A previous study compared the pharmacokinetic profile of rhBMP-2 transplanted with various carriers and found that most transplanted rhBMP-2 were generally released from the implantation site within 14 days (24). Here, the administration period was designed to be longer than 14 days to evaluate the toxicity of rhBMP-2. In addition, the potential maximum clinical dose of 0.05 mg/kg was set as a low-dose group, and toxicity was evaluated for approximately 0.5 mg/kg, which is a maximum of 10 times the dose. Consequently, confirming a sufficient safety margin for the clinical dose from this study was possible.

Because of the procedure, testing the effect of rhBMP-2 implanted by biomaterial carriers from low to high doses is challenging (24). Generally, sufficient animal testing is necessary to determine the appropriate dose of a drug. In nonclinical trials for rhBMP-2, the safety margin of rhBMP-2 cannot be accurately determined since it is used with carriers. Here, the effect of rhBMP-2 was compared at various doses, including a dose of 0.50 mg/kg/day, which is 10 times higher than the potential maximum human clinical dose, to determine a sufficient safety margin of rhBMP-2.

Tail swelling and bending observed in the rats appeared to be local reactions to rhBMP-2 administration, and no toxic effect of rhBMP-2 was observed. The severity of the swelling, bending, and color changes of the tail increased dose-dependently. Two male rats were euthanized because of the severe reaction in the tail, which made the intravenous administration difficult. These changes were not considered adverse because they were limited to the injection sites and were the common findings in laboratory rats receiving tail vein injections. Although rhBMP-2 circulated systemically through the bloodstream after intravenous injection, no additional clinical sign was observed besides the local swelling and bending of the tail. Systemic circulation of rhBMP-2 in rats was confirmed in our previous pharmacokinetic evaluation, and the mean terminal half-life (t1/2) of rhBMP-2 was identified as 6.58 min at 1 mg/kg dose (data not shown). Therefore, this appeared to be a drug reaction, which was limited to the area of administration. Furthermore, previous studies have shown that side effects were locally induced at the site of BMP-2 administration, and no systemic side effects were observed, which was consistent with our results (25, 26).

However, the accumulated damage to the tail may cause discomfort as the dosing times and observation period increase. Therefore, it would be reasonable to assume that it slightly lowered the mean body weight. According to J. Calvez *et al*., the acute local damage to the tissues of the rats caused stress, which can reduce food consumption and consequently lower their body weight (27). However, since the effect was not life-threatening, it was considered that reproductive performance and fertility were not affected.

A previous study showed that rhBMP-2 promotes mammalian oogenesis and folliculogenesis (19). However, in this study, the mean weights of the ovary and oviduct, which are related to reproduction in females, were significantly reduced at the highest rhBMP-2 concentration (0.5 mg/kg/day) without any effects on the reproductive performance (Table III). Therefore, these changes were not considered an adverse effect.

Since rhBMP-2 is not administered daily in clinical studies, the dose and injection interval in this study are inconsistent with those practiced in clinical settings. Therefore, the organs are more strongly exposed to rhBMP-2 because rhBMP-2 is easily distributed throughout the body when administered alone (28). Nevertheless, no adverse effects on reproduction and fertility were observed. Furthermore, a previous study reported no systemic adverse effects when *E.coli*-derived rhBMP-2 was intravenously administered daily with a maximum concentration of 0.50 mg/kg/day to male and female SD rats for 2 weeks (29).

## Conclusions

Overall, this study’s results indicated that the intravenous administration of *E. coli*-derived rhBMP-2 into male and female rats during the pre-mating and early gestation periods has no significant adverse effect on fertility and reproductive performance. Therefore, these results provide novel toxicology information on *E. coli*-derived rhBMP-2 as a therapeutic protein. However, since this study was conducted in rats, its toxicity on fertility in human beings remains unclear; therefore, further research is required to assess whether rhBMP-2 does not affect fertility in women of childbearing age as well as men.

## Data Availability

All data generated or analyzed during this study are included in this published article.
